# Storage of *Euschistus heros* (Fabricius, 1794) Eggs for Biological Control with *Telenomus podisi* Ashmead, 1851 in Open Fields in Brazil

**DOI:** 10.1007/s13744-026-01380-z

**Published:** 2026-03-13

**Authors:** Mikaela Terra Souza Barreto, Carolina Tieppo Camarozano, Marília Corrêa de Melo, Aloísio Coelho Junior, Lessando Moreira Gontijo, Tamara Akemi Takahashi, José Roberto Postali Parra

**Affiliations:** 1 Dept of Entomology and Acarology, Luiz de Queiroz” College of Agriculture, University of São Paulo (USP), Piracicaba, São Paulo, Brazil; 2 Entomology and Nematology Department, University of Florida, Steinmetz Hall, Gainesville, FL USA

**Keywords:** Mass rearing, Egg parasitoids, Quality control, Stink bug

## Abstract

A successful augmentative biological control program requires continuous and large-scale production of high-quality natural enemies, which depends on the development of appropriate storage techniques for these organisms and their hosts. In this study, we stored *Euschistus heros* (Fabricius, 1978) eggs at low temperatures, to determine the most viable storage condition and the duration for which these eggs can be stored without reducing acceptance by the parasitoid *Telenomus podisi* Ashmead, 1893, as well as the quality of its progeny (individuals to be released in the field). Egg storage was evaluated under three conditions: a conventional freezer (–15°C), an ultra-low temperature (ULT) freezer (–80°C), and liquid nitrogen (–196°C) for a period of 12 months. Parasitism of eggs stored under these three conditions was assessed at different intervals. The parasitoid parental (F_0_) generation was evaluated for parasitism, egg-to-adult development time, emergence, and sex ratio. In the progeny (F_1_), parasitism, emergence, and longevity in newly laid *E. heros* eggs were assessed. The ULT freezer and liquid-nitrogen storage conditions resulted in the highest parasitism rates of *T. podisi* in both the parental generation and its progeny. Storage in a conventional freezer was less suitable for parasitism and the viability of *T. podisi*. The results confirm that *E. heros* eggs can be stored in a ULT freezer or liquid nitrogen while maintaining their quality, thereby enabling production and storage during the off-season.

## Introduction

Brazilian grain production reached 299 million tons in 2023, with soybean being one of the key crops contributing to the country's agribusiness success (IBGE 2024). Soybean are cultivated over approximately 47 million hectares in the country (USDA 2025); and the main pest affecting this crop is the brown stink bug *Euschistus heros* (Fabricius, 1978) (Hemiptera: Pentatomidae). Surveys conducted in soybean fields indicate that more than 80% of the stink bug pests recorded belong to this species, which is consistently reported as the most abundant pentatomid associated with soybean crops in Brazil (Bueno et al. 2015; Panizzi et al. 2022; Saldanha et al. 2024). Infestations of *E. heros* cause significant reductions in yield and grain quality (Corrêa-Ferreira and Panizzi 1999; Bueno et al. 2015, 2024), with yield losses reaching up to 30% under high infestation levels (Mais Soja 2024) and economic losses attributed to stink bug complex damage in soybean production estimated at approximately USD 600 million annually (Osaki et al. 2021).

Currently, control of this pest relies on agrochemicals, although a natural enemy, the parasitoid wasp *Telenomus podisi* Ashmead, 1893 (Hymenoptera: Scelionidae), has demonstrated control effectiveness comparable to or even superior to chemical methods (Peres and Corrêa-Ferreira 2004; Queiroz et al. 2018; Silva et al. 2018; Bueno et al. 2020). This parasitoid is already being used on approximately 100,000–200,000 hectares of soybean crops (Parra and Coelho 2022). However, one of the major bottlenecks for large-scale implementation is the availability of host eggs for parasitoid multiplication during the peak occurrence of the target pest. In this context, considering that soybean cultivation in Brazil exceeds 47 million hectares, the area currently covered by this biological control strategy remains marginal in relation to the total soybean growing area.

The limited use of this biological control agent is due to the unavailability of the natural enemy, as there are no mass-rearing systems capable of supplying farmers for widespread parasitoid release, restricting applications to small areas. To date, there is no alternative host or *in-vitro* production of *E. heros* eggs. Although other pentatomid species may be used as alternative hosts for egg production, such as *Diceraeus melacanthus* Dallas, 1851(Hemiptera: Pentatomidae), life-table fertility parameters clearly indicate the superiority of *E. heros* for this purpose (Rodrigues et al. 2023; Souza 2023).

From an applied and industrial perspective, the lack of long-term host egg storage protocols represents a major limitation for scaling biological control programs based on *T. podisi*. Large-scale production requires not only high biological quality but also operational flexibility to synchronize parasitoid availability with pest outbreaks across regions (van Lenteren and Tommasini 2003). Long-term egg storage is essential to ensure a continuous, reliable supply of parasitoids and to optimize production logistics, supporting the industrial viability of *T. podisi* for large-scale biological control.

Additionally, further studies on *E. heros* egg storage techniques are essential to ensure the continuous production of *T. podisi* the year. The storage of *E. heros* eggs at low temperatures, including ultra-low temperature (ULT) freezers and liquid nitrogen, for subsequent parasitism by *T. podisi* has been reported (Favetti et al. 2014; Silva et al. 2019). However, no studies to date have evaluated storage periods exceeding six months, leaving a critical gap in our understanding of the feasibility of long-term egg preservation. Extending storage duration is particularly important in the context of biological control programs, which often involve large treated areas and require the release of high numbers of parasitoids (at least 18,000 individuals per hectare) (Brasil, MAPA 2019; Weber et al. 2022), thereby demanding a substantial and continuous supply of host eggs to sustain large-scale parasitoid production.

Moreover, it is essential to understand how storage duration and methods influence not only the parental generation but also the progeny, as transgenerational effects could enhance or compromise the effectiveness of biological control in the field. Nevertheless, further research is needed since low temperatures and exposure duration may affect reproduction over successive generations (Kivan and Kilic 2005; Mahmoud and Lim 2007; Molaei et al. 2015).

Thus, the present study investigated whether the storage of *E. heros* eggs under different storage methods (conventional freezer at −15°C, ultra-low temperature freezer at −80°C, and liquid nitrogen at −196°C) allows long-term preservation without compromising their acceptance for parasitism or the biological quality of the parasitoid *T. podisi,* both in the parental generation and in its progeny, thereby enabling continuous and large-scale production of the parasitoid for biological control programs and contributing to the operational and industrial scalability of *T. podisi* based technologies in soybean agroecosystems.

## Material and Methods

### Insect Rearing

*Euschistus heros* eggs used in this study were obtained from the rearing colony maintained at the Insect Biology Laboratory of the Department of Entomology and Acarology at USP/ESALQ. The colony was initiated with eggs sourced from the rearing facility of the Brazilian Agricultural Research Corporation (EMBRAPA) Soybean, Londrina, Paraná. The stink bugs were maintained according to the method of Rodrigues (2020), using a natural diet consisting of green bean pods (*Phaseolus vulgaris* L.) and peanuts (*Arachis hypogaea* L.).

*Telenomus podisi* females used in the experiments were obtained from the rearing colony of the Insect Biology Laboratory at the Department of Entomology and Acarology at USP/ESALQ. *E. heros* eggs at 24 h of development were placed in a Petri dish (10 × 2 cm) and offered to adult *T. podisi*, which were kept inside a polyethylene bag and fed with droplets of pure honey. The parasitoids were maintained in rooms with controlled temperature at 25 ± 2°C, relative humidity 70 ± 10%, and a photophase of 14 h. Parasitism was allowed for 24 h; after this period, the Petri dish was removed from the polyethylene bag and kept closed in the same room. One day before emergence, the dish was placed in a new polyethylene bag containing droplets of pure honey for adult feeding.

### Effect of storage on the Parental Generation of *Telenomus podisi*

To determine the optimal storage temperature and duration, 1,500 *E. heros* eggs, up to 24 h old, were stored monthly for 12 months, totaling 18,000 eggs. Three storage methods were assessed: conventional freezer (–15 ± 5°C), ultra-low temperature (ULT) freezer (–80 ± 5°C), and liquid nitrogen (–196 ± 5°C). For conventional freezer and ULT freezer storage, *E. heros* eggs were placed in Falcon tubes (15 mL) and then stored under the respective conditions. Eggs designated for liquid nitrogen storage were placed in aluminum foil envelopes, sealed with masking tape, and then stored in liquid nitrogen inside extraction canisters.

After 12 months of storage using the three methods, the experiment began, evaluating all three storage methods over the 12-month period. The treatments were compared to a control group consisting of freshly laid *E. heros* eggs.

Each treatment consisted of 15 replicates, each consisting of a previously mated *T. podisi* female, which was isolated 48 h after emergence and kept in a glass tube (8 cm × 2 cm) containing a droplet of pure honey as a food source and sealed with PVC plastic film. Each *T. podisi* female was provided with 40 eggs stored under different methods and durations, separately for each treatment (storage method: conventional freezer, ultra-low temperature freezer and liquid nitrogen and period: 1 up to 12 months). In the control treatment, 40 freshly laid *E. heros* eggs were also offered. The eggs were offered loose inside the glass tube, which was kept in a horizontal position to prevent overlap.

The tubes containing the females and eggs were kept for 24 h in climate-controlled BOD chambers at 25 ± 2°C, 70 ± 10% relative humidity, and a photophase of 14 h. After 24 h the females were removed from the tubes, and the eggs were kept in their respective climate-controlled chambers.

The eggs were observed individually, recording the number of parasitized eggs and parasitized eggs with an emergence hole to determine parasitism and emergence rates. The duration of the egg-to-adult period was also evaluated to assess the impact of storage. The number of females and males that emerged daily was recorded to determine whether storage affected the wasps' sex ratio. Sex differentiation was based on antenna morphology, with males having filiform antennae and females having clavate antennae (Johnson 1984). From these F1 adults, new females were selected for the next phase of the experiment.

### Effect of Storage on *Telenomus podisi* Progeny

Fifteen females from the treatments, representing 1, 4, 6, 9, and 12 months of the three storage methods, were mated and then placed in individual glass tubes, following the same method as the previous experiment. However, in this experiment, the females were not removed from the tubes after 24 h.

Daily, 40 fresh (unstored) *E. heros* eggs were offered to the *T. podisi* females in each treatment; after 24 h, the eggs were removed and new eggs were offered. This method was maintained until the females died. The eggs removed daily were placed in new glass tuber, sealed with PVC plastic film, and kept under the same temperature and humidity conditions as the first phase. This allowed for the evaluation of the parasitism, emergence, and longevity of the F1 generation females.

### Statistical Analysis

All analyses were carried out in the R statistical software (R Core Team 2024). Analyses of variance using general linear models were performed to assess the effects of various storage durations on each reproductive parameter, relative to the control (time zero). These analyses were conducted separately for each of the three egg preservation methods. The aim was to compare how storage time influences reproductive outcomes, accounting for differences in preservation techniques. The models were designed to test whether the changes observed in reproductive parameters were statistically significant across different time points compared to the control group.

Additionally, to investigate the potential structuring and grouping of the parasitoid emergence data in relation to the duration of egg storage and preservation methods, a hierarchical cluster analysis was performed using Ward’s method in R. This was carried out separately for the parasitoid emergence data for each of the F_0_ and F_1_ generations. In this analysis, a Euclidean distance metric was used to calculate the distance matrix among the observations. This metric was chosen for its effectiveness in measuring similarities in continuous data. Ward's method (Ward.D2) was employed for the hierarchical clustering. This method minimizes the total within-cluster variance, leading to more-compact and well-separated clusters. Last, a dendrogram was created to visually represent the hierarchical structure of the clusters. This helped in determining the optimal number of clusters by examining the heights of the branches. All analyses were conducted separately for data from either the F_0_ or F_1_ generation of *T. podisi* following egg preservation.

## Results

### Effect of Egg Storage on the Parental Generation of *Telenomus podisi*

**Fig. 1:**
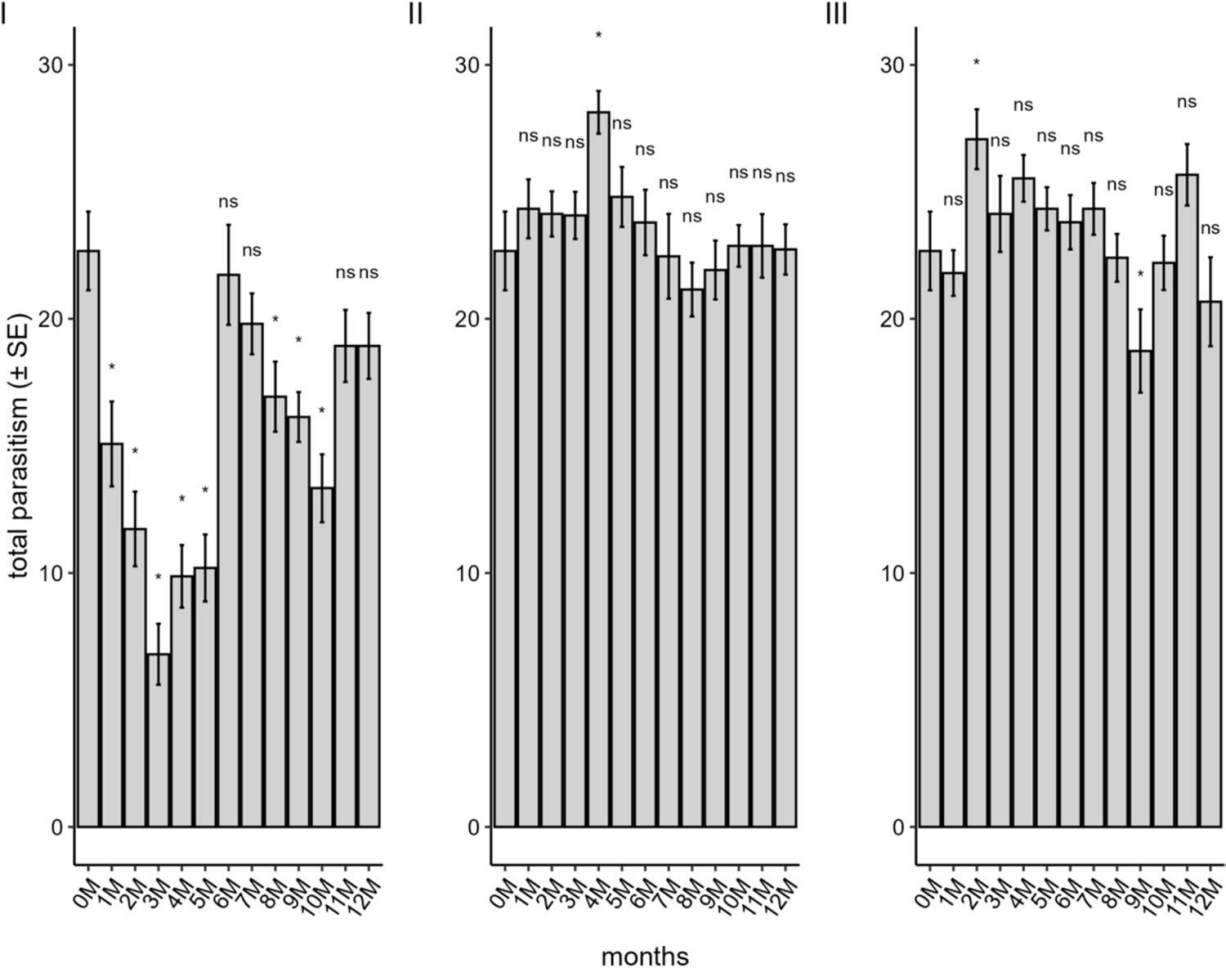
Parasitism of *Telenomus podisi* in the parental generation in *Euschistus heros* eggs stored in a conventional freezer (**I**), liquid nitrogen (**II**), and an ultra-low temperature freezer (**III**) compared to the control (0 M = time zero) (Temperature: 25 ± 1°C, RH: 70 ± 10%, and photophase: 14 h). *Indicates statistical difference compared to control

The parasitism rate of the parental generation (F_0_) was influenced by the storage method and duration, with significant differences observed in the conventional freezer (F_12;182_ = 12.21; *P* < 0.001), liquid nitrogen (F_12;179_ = 1.94; *P* = 0.03), and ULT freezer (F_12;182_ = 3.30; *P* < 0.001) (Fig. 1). Overall parasitism was highest in eggs stored in liquid nitrogen and in a ULT freezer, which showed similar mean parasitism rates (59.18 and 58.47%), values comparable to those observed in the control treatment (fresh eggs: 56.67%). In contrast, eggs stored in a conventional freezer exhibited a lower parasitism rate, averaging 37.39%.

In the conventional freezer treatment, the mean number of parasitized eggs showed considerable variation, with values as low as 6.8 eggs after three months of storage and reaching 21.73 eggs at six months of storage. However, after this period, the mean number of parasitized eggs declined to 17.34 eggs, corresponding to a parasitism rate of 43.36%. In the ULT freezer treatment, the mean number of parasitized eggs remained relatively constant, with values consistently above 20 parasitized eggs (averaging approximately 62% parasitism). A slight reduction was observed at the ninth month of storage (18.73 eggs): however, parasitism levels returned to the aforementioned mean in the remaining storage periods. In the liquid nitrogen treatment, parasitism varied the least among treatments, with the lowest value (21.73 eggs) recorded at the eighth month of storage. Nevertheless, the overall mean remained close to 24 parasitized eggs across all storage durations.

**Fig. 2:**
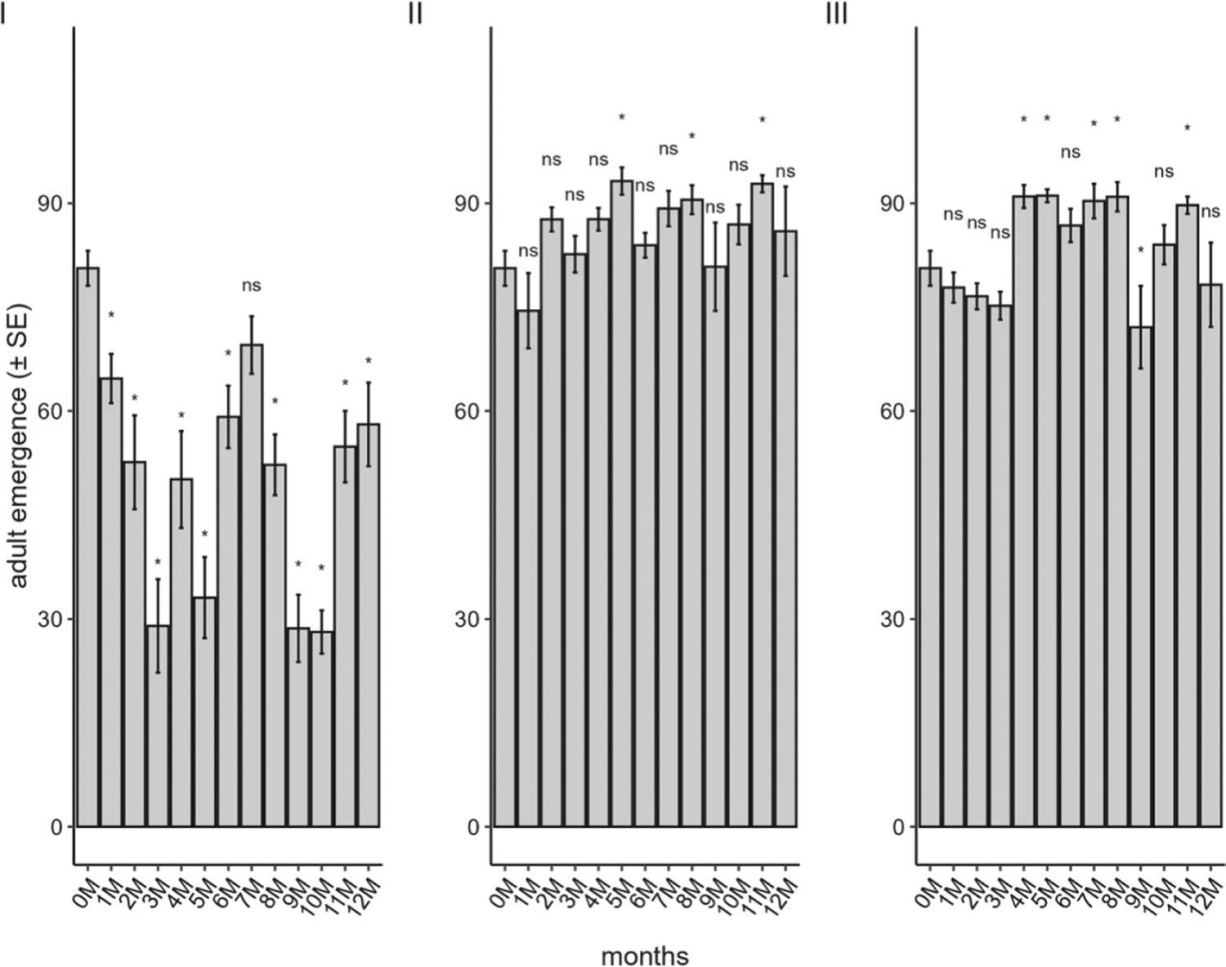
Emergence of *Telenomus podisi* in the parental generation in *Euschistus heros* eggs stored in a conventional freezer (**I**), liquid nitrogen (**II**), and an ultra-low temperature freezer (**III**) compared to the control (0 M = time zero) (Temperature: 25 ± 1°C, RH: 70 ± 10%, and photophase: 14 h). *Indicates statistical difference compared to control

The storage method influenced the emergence of *T. podisi* when eggs were stored in a conventional freezer (F_12;182_ = 10.54; *P* < 0.001), liquid nitrogen (F_12;179_ = 2.39; *P* < 0.001), and ULT freezer (F_12;182_ = 5.21; *P* < 0.001) (Fig. 2). The emergence rate of *T. podisi* from *E. heros* eggs stored in a conventional freezer was in general below 50% with the highest emergence rate recorded at the seventh month of storage (69.52%), and the lowest value at the ninth month of storage (28.13). In contrast, eggs stored in the ULT freezer and liquid nitrogen exhibited consistently high emergence rates, with overall means of 82.21% and 86.30%, respectively, both exceeding the control treatment (80.62%). Although some storage periods differed significantly from the control, emergence rates remained above the reference average in most cases, except for eggs stored for nine months in the ULT freezer, which showed an emergence rate of 72.10%.

**Fig. 3:**
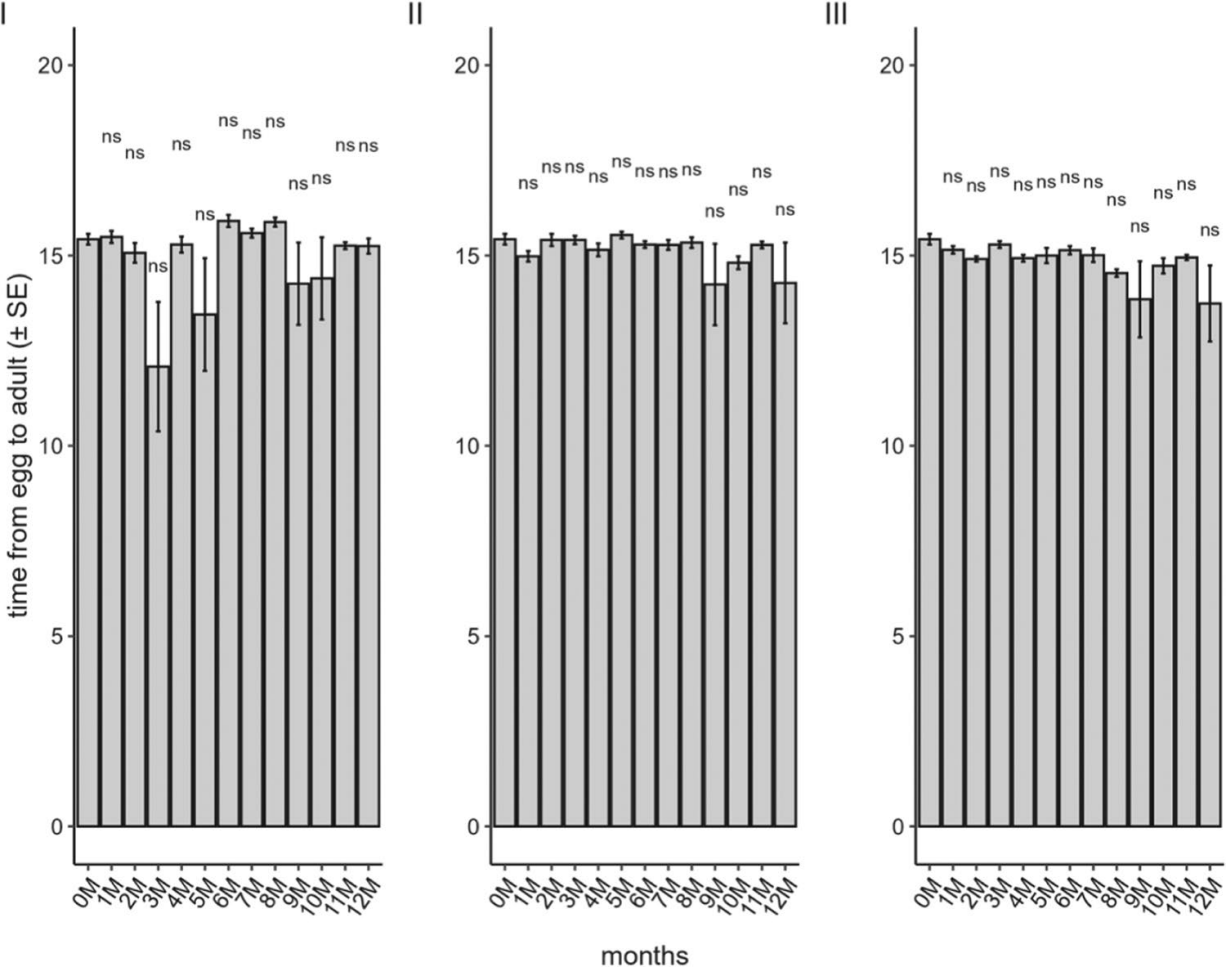
Egg-adult period of *Telenomus podisi* in the parental generation in *Euschistus heros* eggs stored in a conventional freezer (**I**), liquid nitrogen (**II**), and an ultra-low temperature freezer (**III**) compared to the control (0 M = time zero) (Temperature: 25 ± 1°C, RH: 70 ± 10%, and photophase: 14 h). *Indicates statistical difference compared to control

The duration of the egg-to-adult period of *T. podisi* was not affected when stored in a conventional freezer (F_12;182_ = 1.23; *P* = 0.26), ULT freezer (F_12;182_ = 1.49; *P* = 0.13), or liquid nitrogen (F_12;179_ = 0.89; *P* = 0.56). In all treatments, the parasitoids completed their development in approximately 15 days (Fig. 3).

**Fig. 4:**
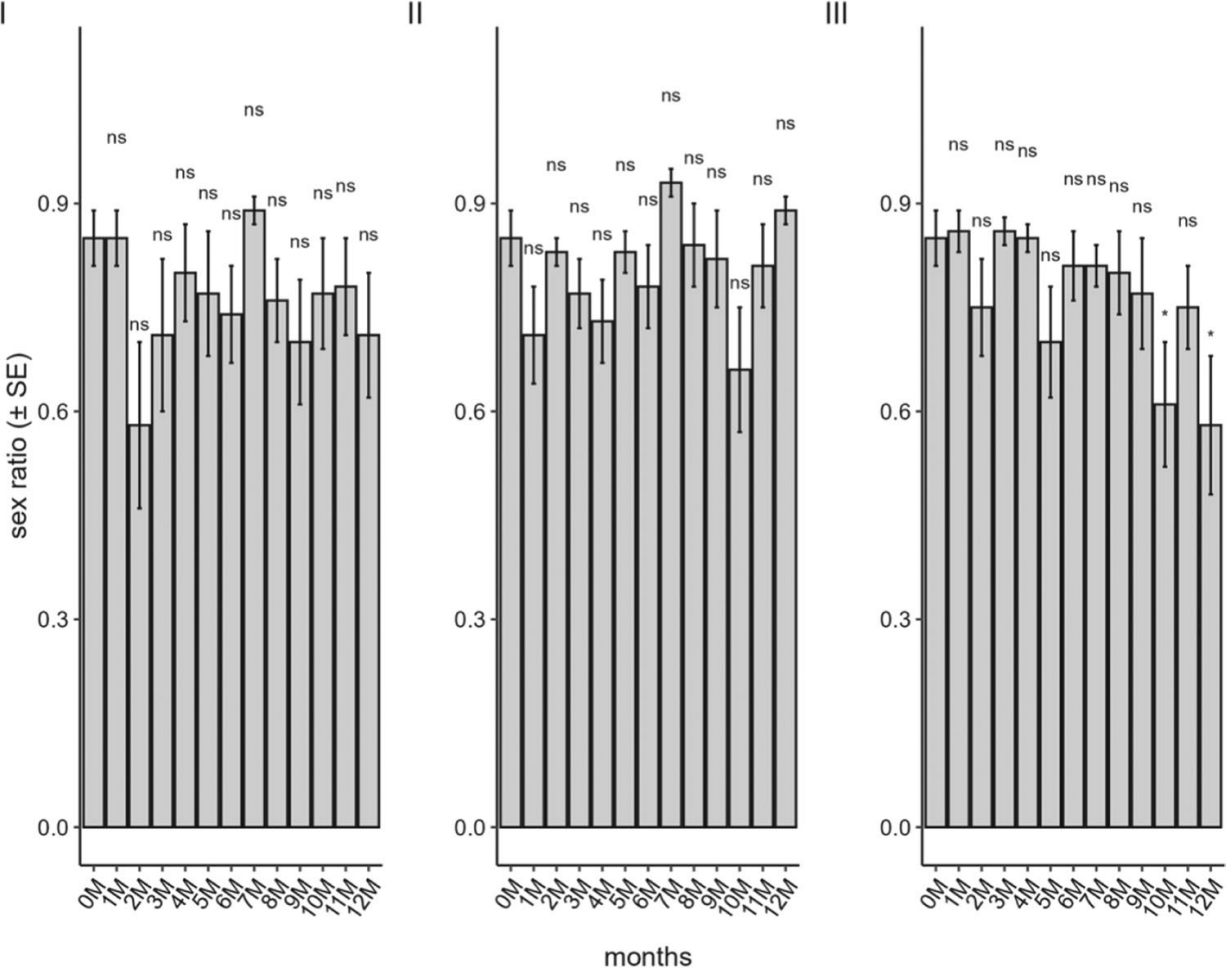
Sex ratio of *Telenomus podisi* in the parental generation in *Euschistus heros* eggs stored in a conventional freezer (**I**), liquid nitrogen (**II**), and an ultra-low temperature freezer (**III**) compared to the control (0 M = time zero) (Temperature: 25 ± 1°C, RH: 70 ± 10%, and photophase: 14 h). *Indicates statistical difference compared to control

The sex ratio of the progeny was affected by the storage periods in the ULT freezer (F_12;182_ = 2.22; *P* = 0.01), with ratios observed at ten and 12 months of storage being 0.62 and 0.54, respectively, while the value for non-stored eggs was 0.85 (Fig. 4). The conventional freezer (F_12;182_ = 1.08; *P* = 0.37) and liquid nitrogen (F_12;179_ = 1.49; *P* = 0.12) methods did not affect the sex ratio, with a ratio of 0.93 observed in eggs stored in liquid nitrogen for seven months.

**Fig. 5:**
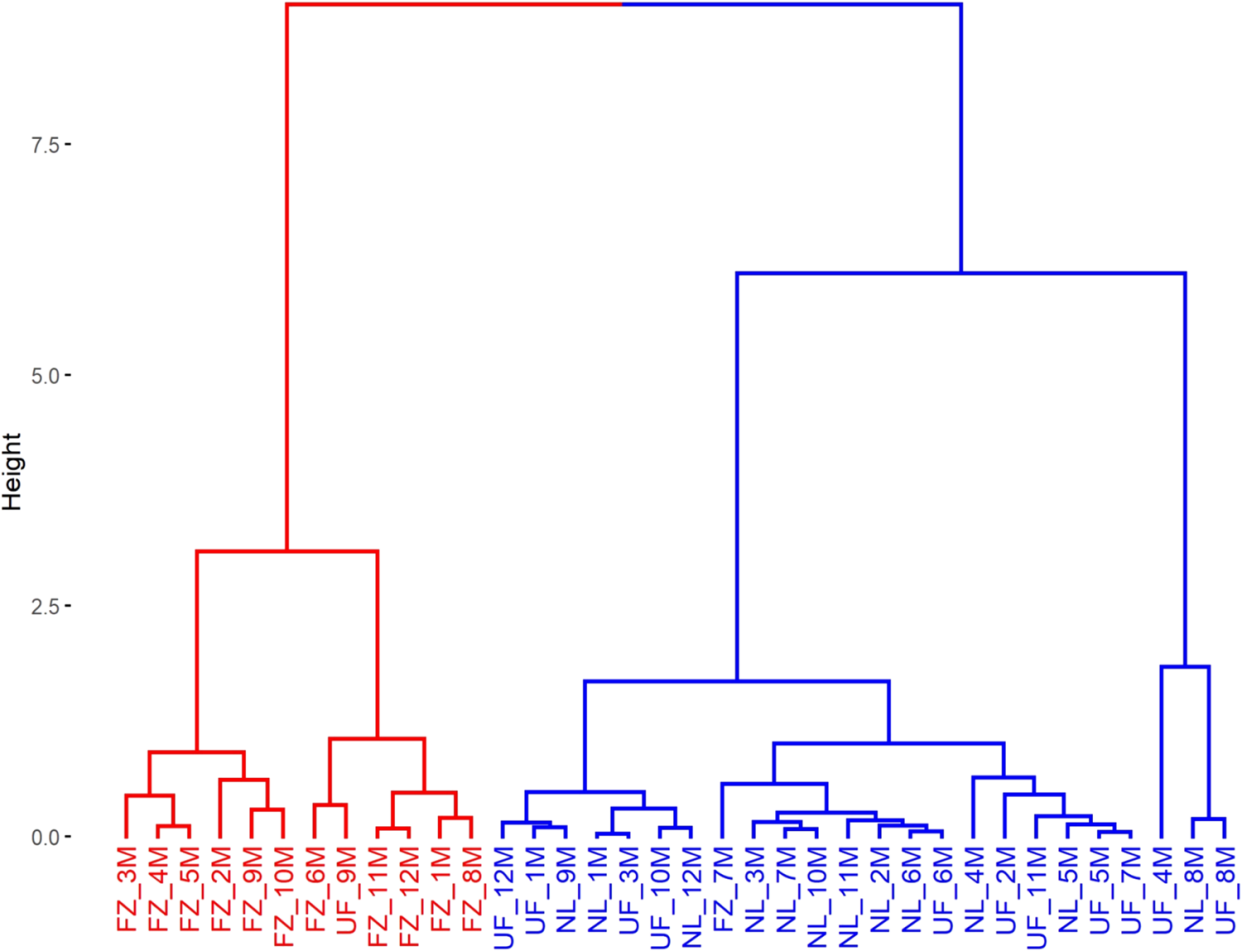
Dendrogram of the parasitism and viability parameters of *Telenomus podisi* in the parental generation in *Euschistus heros* eggs stored in a conventional freezer (FZ), liquid nitrogen (NL), and an ultra-low temperature freezer (UF)

The analysis revealed a distinct clustering in the F_0_ parasitoid emergence data, which separated into two groups: one comprising eggs stored in a conventional freezer and the other consisting of eggs stored in a ULT freezer or in liquid nitrogen (Fig. 5). In contrast, the F_1_ parasitoid emergence data did not show clear clustering based on the different egg storage methods. These findings suggest that the ULT freezer or liquid nitrogen storage methods effectively preserve host eggs, ensuring optimal emergence rates for the F_0_ generation without detrimental effects.

### Effect of Storage on *Telenomus podisi* Progeny

**Fig. 6:**
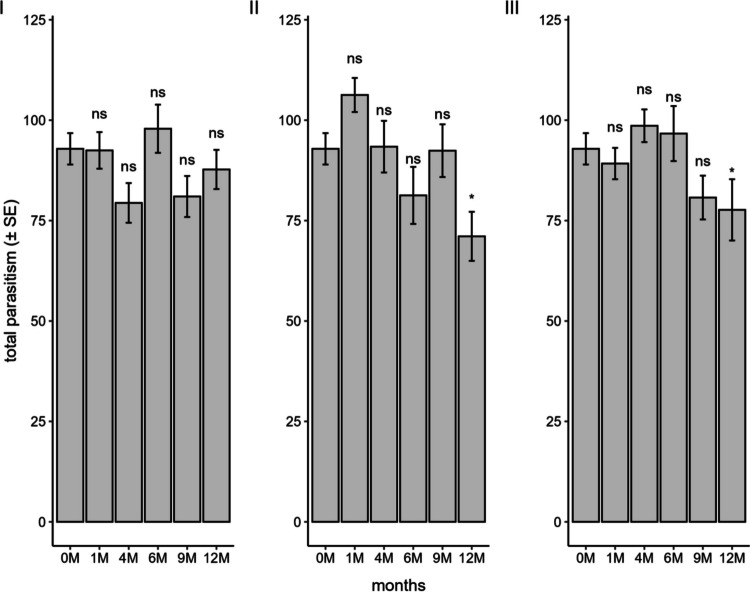
Parasitism of *Telenomus podisi* offspring in the F_1_ generation in *Euschistus heros* eggs stored in a conventional freezer (**I**), liquid nitrogen (**II**), and an ultra-low temperature freezer (**III**) compared to the control (0 M = time zero) (Temperature: 25 ± 1°C, RH: 70 ± 10%, and photophase: 14 h). *Indicates statistical difference compared to the control

The parasitism of the progeny developed from stored eggs was not influenced by the storage method and duration in the conventional freezer (F_5;84_ = 2.15; *P* = 0.07), with cumulative parasitism remaining above 75 eggs in all months (Fig. 6). Only storage for 12 months in liquid nitrogen (F_5;84_ = 4.23; *P* < 0.001) and the ULT freezer (F_5;84_ = 2.40; *P* = 0.04) was detrimental to the F_1_ generation. However, despite the statistical difference observed, for both methods, females were able to parasitize more than 70 eggs during their lifetime, a high number for the species.

**Fig. 7:**
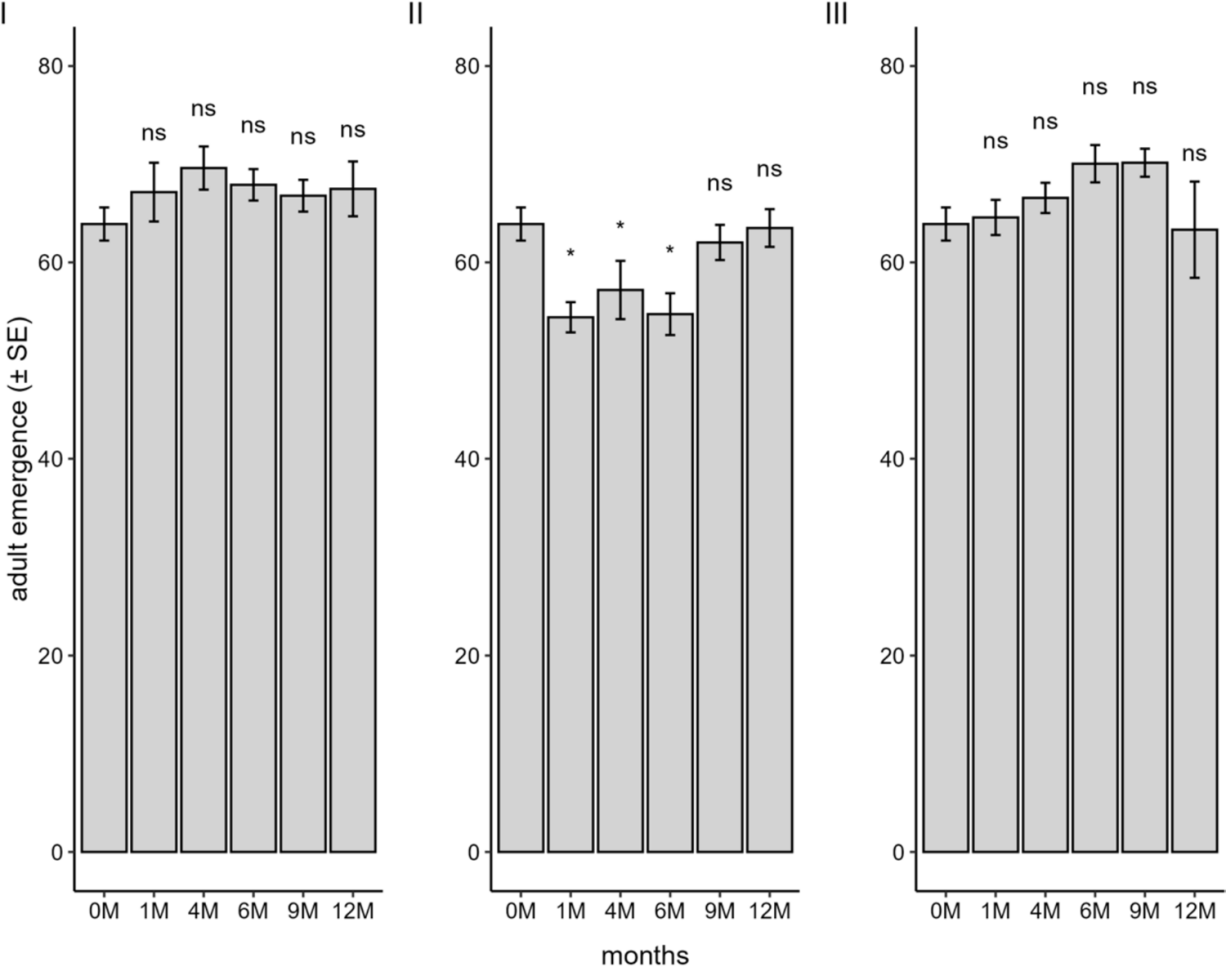
Emergence of *Telenomus podisi* offspring in the F_1_ generation in *Euschistus heros* eggs stored in a conventional freezer (**I**), liquid nitrogen (**II**), and an ultra-low temperature freezer (**III**) compared to the control (0 M = time zero) (Temperature: 25 ± 1°C, RH: 70 ± 10%, and photophase: 14 h). *Indicates statistical difference compared to the control

The emergence of the progeny that developed from eggs stored in a conventional freezer (F_5;84_ = 0.70; *P* = 0.62) and ULT freezer (F_5;84_ = 1.49; *P* = 0.21) was not affected when compared to the control, with all values being above 60% (Fig. 7). However, for liquid nitrogen (F_5;84_ = 4.51; *P* = 0.001), the treatments of one, four, and six months of storage were below 57% compared to non-stored eggs.

**Fig. 8:**
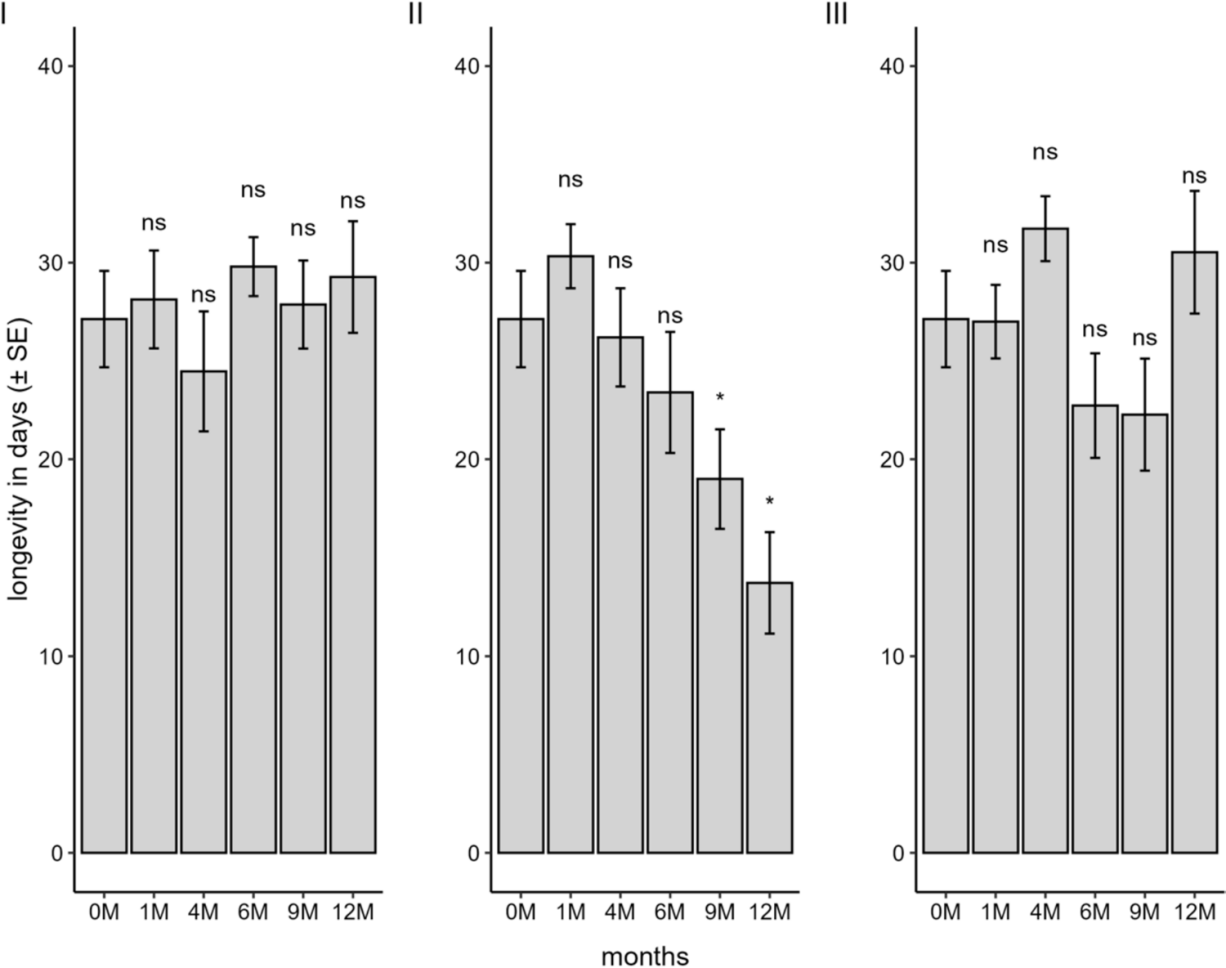
Longevity of *Telenomus podisi* offspring in the F_1_ generation in *Euschistus heros* eggs stored in a conventional freezer (**I**), liquid nitrogen (**II**), and an ultra-low temperature freezer (**III**) compared to the control (0 M = time zero) (Temperature: 25 ± 1°C, RH: 70 ± 10%, and photophase: 14 h). *Indicates statistical difference compared to the control

The longevity of females that developed from stored eggs was not influenced by storage in a conventional freezer (F_5;84_ = 0.79; *P* = 0.55) and ULT freezer (F_5;84_ = 2.44; *P* = 0.04). In these two methods, females from all storage periods lived for more than 20 days (Fig. 8). In liquid nitrogen, a significant difference was observed (F_5;84_ = 5.87; *P* < 0.001) between the periods, with a notable reduction in female longevity of 30% and 45% at 9 and 12 months, respectively, compared to the control.

## Discussion

The large-scale implementation of *T. podisi* in soybean cropping systems is primarily constrained by the continuous availability of high-quality host eggs for mass rearing. In the present study, we demonstrate that long-term storage of *E. heros* eggs, particularly under ultra-low temperature (−80°C) and liquid nitrogen (−196°C) conditions, is feasible for periods of up to 12 months without substantially compromising parasitoid parasitism, emergence, or overall biological quality. These findings represent an important advance toward overcoming one of the major operational bottlenecks limiting the expansion of *T. podisi* releases in Brazil.

In recent years, considerable efforts have been devoted to defining optimal release rates, release intervals, and the soybean phenological stage for the application of *T. podisi* and these parameters are now relatively well established (Weber et al. 2022; Oliveira et al. 2024). However, such advances must be accompanied by strategies that ensure a reliable and continuos supply of host eggs for parasitoid production. The development of efficient mass-rearing systems for *E. heros*, including the automation of rearing procedures and the implementation of effective egg storage techniques, is therefore essential to meet the demands of large-scale Brazilian agriculture. In this context, low-temperature storage, particularly for long-term preservation, emerges as a key component for enabling continuous parasitoid production throughout the year and for buffering unexpected peaks in demand, which are common in a country with continental dimensions such as Brazil (van Lenteren and Tommasini 2003).

Although several studies have reported successful storage of pentatomid eggs at low temperatures (Favetti et al. 2014; Silva et al. 2019; Almeida et al. 2023; Ramos et al. 2025), to our knowledge, this is the first study to evaluate the long-term storage of *E. heros* eggs for periods of up to 12 months. When overall parasitism performance was considered, storage in an ultra-low temperature (ULT) freezer and in liquid nitrogen proved to be considered the most suitable preservation methods. Both treatments resulted in high mean parasitism rates (58.47% and 89.18%, respectively), which is similar those observed in the control treatment using freshly laid eggs (56.67%) In contrast, eggs stored in the conventional freezer exhibited substantially lower parasitism rates (37.39%), indicating that this method is the least suitable for the mass production of *T. podisi*. These findings are partially consistent with previous studies evaluating low-temperature eggs storage in pentatomids. Almeida et al. (2023), for example, assessed different storage methods and egg packaging strategies for *E. heros* and reported that eggs wrapped in aluminum foil and stored in a conventional freezer (−18°C) or in liquid nitrogen (−196°C) remained viable for parasitism for up to 60 days, although with significant variation among storage periods. However, the storage duration evaluated by those authors was limited to 60 days, whereas the present study extends this assessment to substantially longer periods, providing new evidence for the feasibility of long-term egg preservation. Similarly, Silva et al. (2019) reported lower parasitism rates when *E. heros* eggs were stored under ultra-low temperature conditions (−80°C and −196°C) for up to 70 days; in contrast the present results demonstrate that high parasitism levels can be maintained over much longer storage periods when appropriate protocols are applied.

The lower performance in parasitism observed for eggs stored in conventional freezer is likely related to cellular damage caused by ice crystal formation during freezing and thawing processes at moderately low temperatures (–15°C). At such temperatures, intracellular and extracellular ice crystallization may compromise egg tissue, thereby reducing host quality for parasitoid development (Canet 1989). Freezing speed is a critical factor for preservation cellular integrity, as rapid cooling, such as that achieved during vitrification in liquid nitrogen, minimizes ice crystal formation within cellular compartments (Milward-de-Azevedo et al. 2004). This mechanism likely explains the superior parasitism and emergence rates observed for eggs stored under ultra-low temperature and cryogenic conditions in the present study.

Eggs stored in an ultra-low temperature (ULT) freezer or in liquid nitrogen for up to 12 months exhibited emergence rates exceeding 85%, whereas eggs stored in a conventional freezer showed mean emergence values below 50%. These results are consistent with those reported by Silva et al. (2019), who documented significantly lower emergence from *E. heros* eggs stored at −15°C compared with those maintained under ultra-lower temperature and liquid nitrogen conditions, further emphasizing the limited suitability of conventional freezing for long-term egg preservation.

In contrast, the duration of parasitoid development was not affect by any of the storage methods evaluated. This finding is consistent with Oliveira et al. (2024), who reported no differences in the development time of *T. podisi* emerging from fresh eggs or eggs stored in liquid nitrogen under different rearing temperatures. However, Ramos et al. (2025) observed a slight delay of approximately one day in the development of *T. podisi* originating from cryopreserved eggs compared with those developing from fresh eggs. Suggesting that subtle effects on developmental dynamics may occur depending on storage duration and protocol.

Regarding transgenerational responses, only minor reductions were detected in specific biological parameters of the progeny originating from eggs stored in ULT freezer and liquid nitrogen. Nevertheless, considering the extended storage period evaluated (up to 12 months) and the overall biology of the parasitoid, these effects were limited and did not compromise the functional quality of the F1 generation. Similar conclusions were reached by Ramos et al. (2025) who reported no significant differences in parasitism, emergence, sex ratio, or longevity of *T. podisi* females developing from *E. heros* eggs stored in liquid nitrogen, although in that study eggs were preserved for only 30 days. Favetti et al. (2014) also reported high parasitism rates from *E. heros* eggs stored in liquid nitrogen for three and six months; however, the lack of detailed quantitative information regarding egg availability limits direct comparisons with the present results.

It is important to note that long-term egg storage outcomes may be further optimized by refining cryopreservation protocols, particularly thawing procedures prior to egg exposure to parasitoid females. In addition to freezing rate, thawing speed plays a crucial role in preserving biological quality, as rapid thawing in a controlled water bath (35–40°C) can prevent the fusion of microcrystals formed during freezing and reduce damage associated with devitrification (Santos 2000). Such refinements may further enhance egg viability and parasitoid performance following extended storage periods. From an applied perspective, the implications of long-term egg storage are substantial. The recommended release rate of *T. podisi* for the control of *E. heros* is approximately 6,500 parasitoids per hectare, applied in three consecutive releases at weekly intervals, resulting in a total requirement of nearly 19,500 parasitoids per hectare. As *T. podisi* is a solitary parasitoid, this release strategy requires at least one host egg per individual produced. Consequently, implementing biological control across the entire soybean-growing area in Brazil would require a minimum of approximately one billion *E. heros* eggs.

Considering that parasitism viability after 12 months of storage averages approximately 60% for eggs preserved in ULT freezers and liquid nitrogen, but only around 40% for eggs stored in conventional freezers, achieving one billion eggs viable for parasitism would require an increase in egg production of more than 150% when using conventional freezer storage, whereas ULT freezer and liquid nitrogen storage would require an increase of approximately 70%. Specifically, conventional freezing would require the production of approximately 2.5 billion eggs, compared with about 1.67 billion eggs required under ultra-low temperature or liquid nitrogen storage. These differences highlight the importance of incorporating cost–benefit analyses into decision-making processes regarding storage methodologies for large-scale biological control programs.

Overall, our results demonstrate that *E. heros* eggs can be stored in an ultra-low temperature freezer or in liquid nitrogen for up to 12 months without significantly affecting either the parental generation of *T. podisi* produced in the laboratory and released in the field or their progeny. Under favorable field conditions, such progeny may contribute to the persistence and continuity of parasitoid populations, thereby enhancing the effectiveness and sustainability of biological control strategies in soybean agroecosystems.

## Data Availability

The datasets generated and analyzed during the present study are available from the corresponding author upon reasonable request.
